# Diagnosis and Treatment of Female Infertility Is One of the Major Problems in Modern Gynecology

**Published:** 2018-01

**Authors:** Assem AKETAYEVA, Zaituna KHAMIDULLINA, Zhanar AKHMETOVA, Assel BAUBEKOVA, Zaituna KHISMETOVA, Yelena DUDNIK, Zhaina AITBAEVA

**Affiliations:** 1. Dept. of Obstetrics & Gynecology, Medical University JSC, Astana, Kazakhstan; 2. Dept. of Neurology, Medical University JSC, Astana, Kazakhstan; 3. Semey State Medical University, Semey, Kazakhstan; 4. Dept. of Physiopathology of the Name of V.G. Korpacheva, Medical University JSC, Astana, Kazakhstan

## Dear Editor-in-Chief

Female sterility currently remains one of the most pressing challenges in contemporary gynecological practice at the present stage of development (1).

The leading cause of female sterility is tube-peritoneal which rate does not have a downward trend. It primarily related to increase of inflammatory diseases of uterine annexes.

Therefore, the development of effective methods for diagnosis and management of female sterility are among the major tasks in contemporary gynecology. The progress achieved in various areas of science has enabled to take a medical aid to the patients with female sterility to a completely new level. This has become possible by using the latest techniques of examination, namely endoscopic methods using state-of-the-art technical aid (2–4).

In this regard, the issues of fallopian tube diagnostics have a special consideration in development of endoscopic methods in infertile women. During endoscopy, namely the abdominoscopy the state of alvus, ovarian and fallopian tube, evidence of acute and chronic inflammation of endometriosis, uterine fibroid that requires the establishment of necessary treatment were assessed.

This study aimed to determine the state of the fallopian tubes of women with natural sterility in combination with pathologies of reproductive organs. This study was conducted in Gynecology Departments of Perinatal Centers of the Republic of Kazakhstan from 2014 to 2017, on 250 patients. Of them, we examined 122 with natural sterility, 128 with secondary sterility.

Informed consent was taken from the participants and the study was approved by Ethics Committee of the university.

The patients with natural sterility were distributed to three groups:

1. With endometriosis (n=41), average age was equal to 31.26 + 4.89 yr.
2. With uterine fibroid (n=32), average age was equal to 36.25 + 2.92 yr
3. With chronic salpingitis (n=49), average age was equal to 30.30 + 4.41 yr (Table 1).

**Table 1:** Average age of offensive menstrual functions

**Parameter**	**Group 1**	**Group 2**	**Group 3**
Age (yr)	M± m	M± m	M± m
	n =41	n =32	n =49
12–14	13,05±0.83 yr	13.0±0.70 yr	13.07±0.74 yr
15–16	15.6±0.49 yr	15.6±0.48 yr	15.75±0.43 yr

All the patients have passed the routine examination; exclusionary criteria consisted of the presence of an endocrine form and the male factor of infertility.

The obtained data were statistically processed on individual computer using Excel, and SPSS (Version 20, Chicago, IL, USA).

The distinctions between the groups were statistically valid by Fisher’s exact test (*P*=1.0) at (*P*<0.05), by this attribute the groups appeared to be comparable.

The most frequently detectable abnormality in patients with natural sterility combined with chronic salpingitis are the diseases of endocrine system (thyroid disorders −15.38%). By other attributes, there was no statistical discrepancy in Fisher (*P*=0.42) at (*P*<0.05). The analysis of associated gynecological disorders has shown that in Fisher *P*=0.3 at. In such a manner by attributes of surgical service the statistical analysis has shown that according to Fisher *P*=0.01 at (*P*<0.05). The analysis of obtained data has shown that according to Fisher *P*=0.82 at (*P*<0.05).

**Fig. 1:**
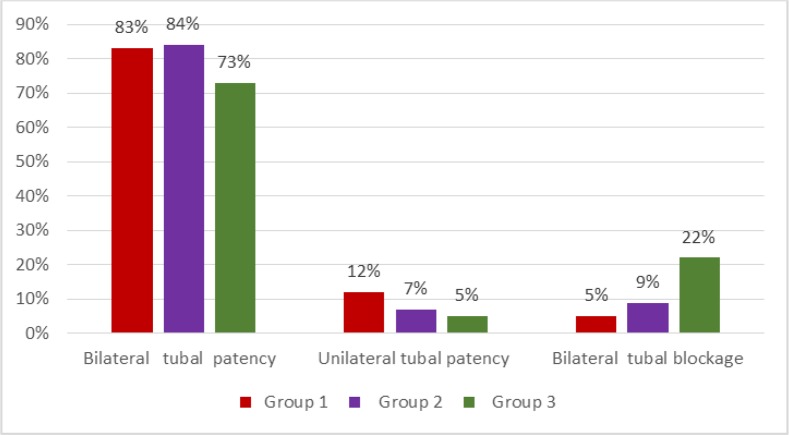
Characteristics of tubal patency on the basis of the data of sonographic hydrotubation of the examined women

Data of sonographic hydrotubation in examined women in compared groups is presented in (Fig. 1).

In such a manner in all three analyzable groups, the majority of cases have turned to be comparable. In view of the presence of patients with natural sterility combined with reproductive diseases, the frequency of fallopian tube abnormality in three groups was equal from 30% to 82.05%, despite its double-sided uterine tubes patency from 73% to 84%.

Therefore, the precise diagnosis establishing is possible with correct observation of algorithm for examination of patients with natural sterility combined with reproductive diseases including the careful examination of complaints, anamnesis, and data of special pelvic examination.

Therefore, at the outpatient stage in the diagnosis of natural sterility in combination with the pathology of the reproductive organs, hysterosalpingography becomes more 5 that are important. The endoscopic interventions shall be performed not with the purpose of diagnosis verification but with medical purpose.
